# A cost-sensitive deep neural network-based prediction model for the mortality in acute myocardial infarction patients with hypertension on imbalanced data

**DOI:** 10.3389/fcvm.2024.1276608

**Published:** 2024-03-19

**Authors:** Huilin Zheng, Syed Waseem Abbas Sherazi, Jong Yun Lee

**Affiliations:** ^1^ Department of Computer Science, Chungbuk National University, Cheongju, Republic of Korea; ^2^ College of Computer Science and Engineering, Guilin University of Technology, Guilin, China

**Keywords:** acute myocardial infarction, mortality prediction, hypertension, deep learning, cost-sensitive learning, threshold moving

## Abstract

**Background and objectives:**

Hypertension is one of the most serious risk factors and the leading cause of mortality in patients with cardiovascular diseases (CVDs). It is necessary to accurately predict the mortality of patients suffering from CVDs with hypertension. Therefore, this paper proposes a novel cost-sensitive deep neural network (CSDNN)-based mortality prediction model for out-of-hospital acute myocardial infarction (AMI) patients with hypertension on imbalanced data.

**Methods:**

The synopsis of our research is as follows. First, the experimental data is extracted from the Korea Acute Myocardial Infarction Registry-National Institutes of Health (KAMIR-NIH) and preprocessed with several approaches. Then the imbalanced experimental dataset is divided into training data (80%) and test data (20%). After that, we design the proposed CSDNN-based mortality prediction model, which can solve the skewed class distribution between the majority and minority classes in the training data. The threshold moving technique is also employed to enhance the performance of the proposed model. Finally, we evaluate the performance of the proposed model using the test data and compare it with other commonly used machine learning (ML) and data sampling-based ensemble models. Moreover, the hyperparameters of all models are optimized through random search strategies with a 5-fold cross-validation approach.

**Results and discussion:**

In the result, the proposed CSDNN model with the threshold moving technique yielded the best results on imbalanced data. Additionally, our proposed model outperformed the best ML model and the classic data sampling-based ensemble model with an AUC of 2.58% and 2.55% improvement, respectively. It aids in decision-making and offers a precise mortality prediction for AMI patients with hypertension.

## 1 Introduction

Cardiovascular diseases (CVDs) are the main type of noncommunicable diseases (NCDs) and account for most NCD deaths (1). It caused approximately 17.9 million deaths in 2019, more than one-third of deaths worldwide (2). Hypertension is one of the primary NCD risk factors and also one of the most critical risk factors for CVDs, also known as high blood pressure (3, 4, 7). It is known as a “silent killer” because the signs and symptoms usually do not occur until hypertension has reached the severe stage (5). In 2015, approximately 1 in 4 males and 1 in 5 females worldwide suffered from hypertension (6). Furthermore, high systolic and diastolic blood pressure is widely known to increase the mortality risk of CVD patients (8, 9). Hence, this paper targets the mortality prediction of AMI patients with hypertension, since many existing research does not mainly focus on CVD patients with hypertension. Regarding disease risk prediction and clinical prognosis for cardiovascular diseases (CVDs) and hypertension, there are generally two main categories of approaches: traditional regression-based and machine learning (ML)-based methods. Conventional regression-based methods, such as the Global Registry of Acute Coronary Events (GRACE) (10), Systematic Coronary Risk Evaluation (SCORE) (11), Thrombolysis in Myocardial Infarction (TIMI) (12), and Framingham Risk Scores (FRS) (13), etc. have been developed for the prediction of CVDs, whereas Cox proportional-hazards regression, Weibull regression, etc. have been used for the hypertension prediction a long time ago (14). However, the conventional regression-based models consider few risk factors and cannot deal with the missing values efficiently, which leads to a lower performance for the mortality prediction of CVD patients. In addition, several ML-based models using support vector machine (SVM), logistic regression (LR), decision tree (DT), random forest (RF), adaptive boosting (AdaBoost), extreme gradient boosting (XGBoost), etc. were also developed for the prediction of CVDs and hypertension, which is better than the traditional regression-based models generally (15–18). Deep learning (DL), one of the stated methods in ML, has advanced significantly in the previous ten years due to its powerful computational capacity (19). It has been used in various domains successfully including healthcare, such as cancer diagnosis (20), heart disease prediction (21, 22), drug response prediction (23), medical image analysis (24–26), etc. In DL techniques, the deep neural network (DNN) is a type of artificial neural network (ANN) that includes multiple hidden layers for the detection of more complex non-linear relationships between the input and output (27). It has shown a strong ability over general ML-based methods in different research. Hence, the DL-based approach is a better choice for predicting the seriousness and mortality in CVD patients with hypertension.

The class imbalance, defined as the skewed class distribution between the majority and minority classes, is also a common issue in the datasets from different domains, especially in medical datasets in which the majority class is the healthy person and the minority class is the patients. Most of the classifiers get biased results for the majority class when analyzing imbalanced data and ignore the minority class data in the highly imbalanced case. Several approaches such as data-level and algorithm-level methods can be applied to address this problem (28). In data-level techniques, various data oversampling and undersampling methods are applied to reduce imbalance levels (18, 29). However, data sampling techniques have some potential limitations. First, it may increase computation costs with unnecessary instances and obscure some potentially valuable data. Second, the data sampling method has the serious limitation of biased selection, which leads to incorrect conclusions. Third, the distribution of various classes is also affected by both undersampling and oversampling (30). In the algorithm-level technique, the cost or weight schema is used to mitigate the bias towards the majority class in the underlying classifiers or its output, which is famous as cost-sensitive learning (31). Compared with data-level techniques, this technique does not require the alteration of the original data distribution as the modified algorithms consider the uneven distribution of classes while training, which results in more accurate performance than data sampling techniques (32). In addition, a simple and straightforward method named threshold-moving has also shown effective results for the class imbalance problem, which moves the decision threshold in the output to make the high-cost samples harder to misclassify (33, 34).

Therefore, this paper proposes a cost-sensitive deep neural network (CSDNN)-based prediction model to forecast the mortality in out-of-hospital AMI patients with hypertension while using the threshold moving technique to improve the performance on imbalanced tabular data. Our research contributions can be outlined as follows: First, a DL method is proposed with a cost-sensitive learning technique to generate an accurate model for the mortality prediction of AMI patients with hypertension. Second, the proposed method with the threshold moving technique shows the efficiency of handling the imbalanced data problem. Third, several classic data sampling-based ensemble models such as balanced bagging (35), balanced RF (36), EasyEnsemble (37), and RUSBoost (38) classifiers which have shown good performance on imbalanced data are utilized to evaluate the performance and robustness of the proposed CSDNN-based mortality prediction model. Finally, the wrapper-based feature selection method, which combines Recursive Feature Elimination (RFE) with a cross-validation strategy for optimal feature selection to speed up all models, has demonstrated performance improvement in the proposed and other models.

The rest of the paper is organized as follows: Section 2 provides an overview of the related work on ML-based disease prediction and the solution of imbalanced medical data. Section 3 introduces the experimental dataset and methods applied in this paper. Section 4 presents the experimental results and discussion. Finally, Section 5 concludes the overall research.

## 2 Related work

### 2.1 Machine learning-based disease prediction

ML techniques have been used to predict various diseases popularly. For example, Sherazi et al. (15) developed the ML-based 1-year mortality prognosis model for 8,227 Korean CVD patients, which showed that the applied ML algorithm improved the performance by 8% over the traditional GRACE model. Chang et al. (16) proposed ML-based prediction models for outcomes of hypertension patients using four classifiers such as DT, SVM, RF, and XGBoost, where their results showed that the XGBoost achieved the best prediction performance. Weng et al. (17) compared ML-based algorithms such as RF, LR, etc. with an established American Heart Association/American College of Cardiology (ACC/AHA) algorithm for the risk prediction of CVD in large-size data with 378,256 patients. The results exhibited that all ML algorithms improved the prediction performance than the baseline ACC/AHA algorithm. In addition, DL techniques have been also widely used in the medical field. Ali et al. (21) proposed an automatic diagnostic system for heart disease prediction based on the DNN. They demonstrated that the proposed method achieved a prediction accuracy of 93.33% and outperformed many other state-of-the-art ML-based methods such as SVM, RF, AdaBoost, etc. Das et al. (22) applied several ML and DL algorithms to detect heart disease using LR, DT, SVM, ANN, etc. In their result, the ANN achieved the best accuracy and was superior to other ML-based approaches.

### 2.2 Solution of imbalanced medical data

Class imbalance often occurs in medical data, where the number of healthy individuals is greater than the number of patients. Various techniques can be used to solve this problem. The first method is the data-level technique. For instance, Zheng et al. (18) applied three types of data oversampling, undersampling, and hybrid sampling techniques to handle the class imbalance problem in patients with CVDs. Their results demonstrated that the proposed ML-based model using the hybrid data sampling method improved the accuracy of the final prediction results. Wang et al. (29) used an adaptive synthetic sampling approach (ADASYN) data oversampling technique to reduce the influence of class imbalance and then designed the RF classifier to predict diabetes. As a result, the method they proposed proved to be effective and superior. Secondly, the technique can also be used at the algorithm level. Mienye et al. (30) implemented various cost-sensitive learning algorithms such as DT, RF, LR, and XGBoost for four medical datasets. Their results showed the effectiveness of cost-sensitive learning in predicting imbalanced medical datasets. Qi et al. (31) proposed a hybrid cost-sensitive ensemble method based on three public datasets from the UCI machine learning repository for heart disease prediction. The results demonstrated that the proposed method could improve the efficiency of diagnosis and reduce the misclassification cost using the cost-sensitive learning strategy. Third, the simple threshold moving method can be applied. Mulugeta et al. (32) used several ML algorithms such as LR, Naïve Bayes, ANN, RF, etc., with the threshold moving technique to predict the risk of graft failure on imbalanced kidney transplant recipients data. The results showed that the data-driven threshold moving technique improved the prediction result from imbalanced data compared to the natural threshold of 0.5.

## 3 Materials and methods

### 3.1 Experimental framework

**Figure 1:**
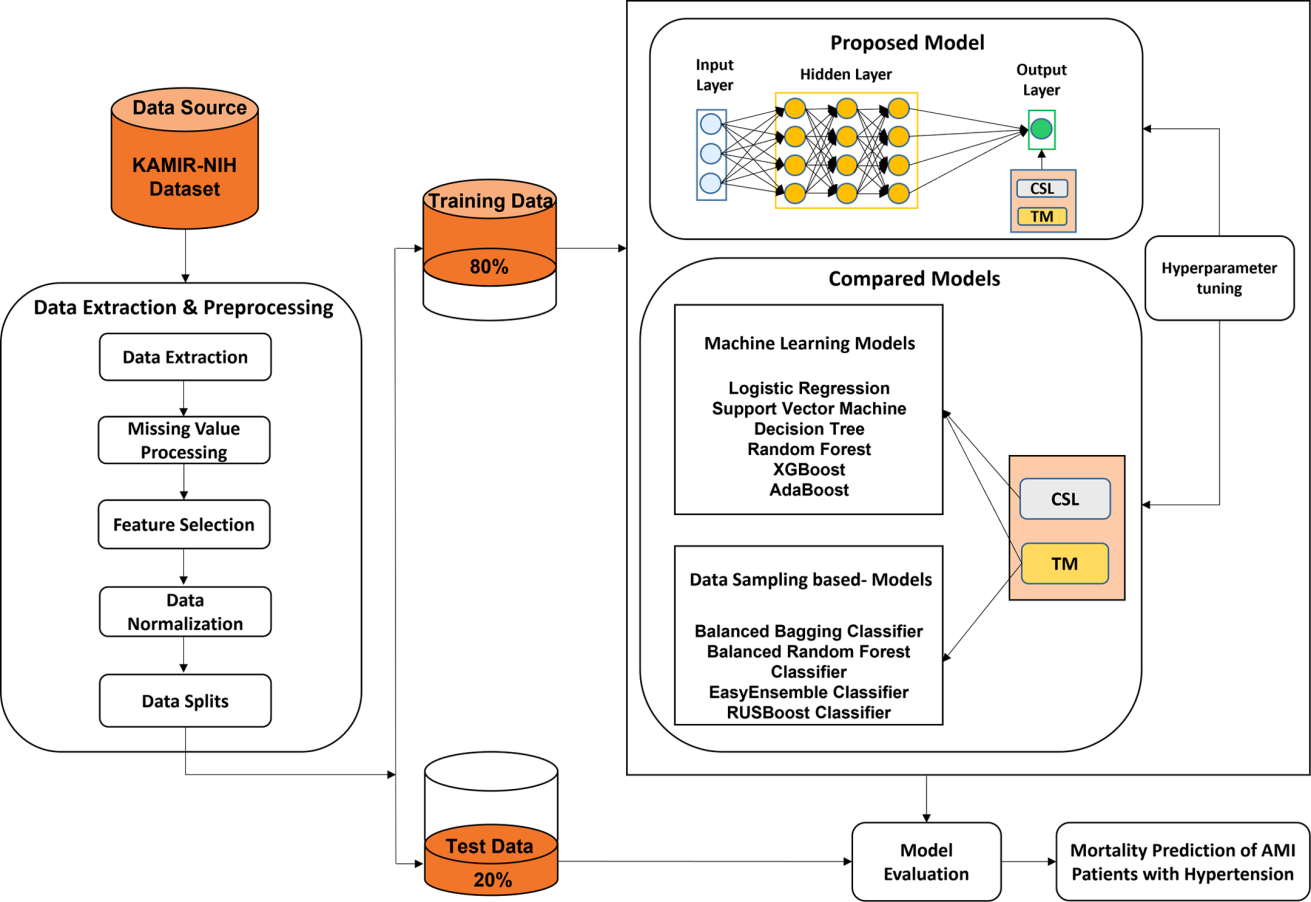
The experimental framework for the prediction of mortality in AMI patients with hypertension.

The experimental framework for mortality prediction in AMI patients with hypertension is shown in Figure 1, which mainly includes three parts: data extraction and preprocessing, predictive model generation, and model evaluation. The mortality of AMI patients is defined as cardiac death and non-cardiac death which is the target feature of this paper. In the first part, we extract the experimental data from the Korean Acute Myocardial Infarction Registry-National Institutes of Health (KAMIR-NIH) dataset (39) and preprocess the data, such as handling the missing values and irrelevant features, normalizing the data, and then splitting the data into training (80%) and test data (20%). In the second part, the proposed CSDNN-based mortality prediction model and several compared models are developed using the training data. Moreover, the hyperparameters are also optimized for each model to get high performance. In the end, the test data is used to evaluate the performance of the proposed model for the mortality prediction of AMI patients with hypertension and also compared with other prediction models.

### 3.2 Data extraction and data preprocessing

KAMIR is the first nationwide, prospective, multicenter registry specially designed to assess patients with AMI (40) in South Korea which is registered with 52 different Korean university hospitals and communities. The experiment in this paper is based on the KAMIR-NIH dataset, which includes 13,104 AMI patients’ records and 550 features with 2-year follow-ups from November 2011 to December 2019 (39). First, the experimental data is extracted from the original dataset for the target, where the total record of 5,602 out-of-hospital AMI patients with hypertension is extracted from the original 13,104 records and excluded the AMI patients' records died at the hospital (Excluded *N* = 504), failed to follow up for 2 years (Excluded *N* = 1,411), and without hypertension (Excluded *N* = 5,587). A total of 64 features are extracted from the KAMIR-NIH dataset, where 1 feature is used as the target variable and the other 63 features are used as the independent variables. The extracted data includes the demographic characteristics, clinical findings, medical history, and laboratory findings which refer to different studies (15, 18, 41, 42), as shown in Table 1. The experimental dataset has a strong representativeness of out-of-hospital AMI patients with hypertension and can be used to design our proposed prediction model for the target.

**Table 1:** The applied features from the KAMIR-NIH dataset.

Type	Features
Categorical (31)	Gender, Chest Pain, Dyspnea, Previous Chest Pain, Killip Class, ECG Use at Admission, ST-change on ECG, Past Medical Diagnoses, Diabetes Mellitus, Dyslipidemia, Previous MI, Previous Angina Pectoris, Previous Heart Failure, Previous Cerebrovascular Disease, History of Smoking, Family History of Heart Disease, Family History of Early Age Ischemic Heart Disease, MI Symptoms, MI ECG Change, MI Imaging, Pre-TIMI Flow of Target Vessel, Post-TIMI Flow of Target Vessel, Initial Diagnosis of STEMI & NSTEMI, Use of Thrombolysis, Thrombolysis Outcome, Use of CAG, CAG Result, ECG Use in Hospital, Final Diagnosis of STEMI & NSTEMI, Discharge Type of Patient, Mortality in 24 Month
Continuous (43)	Age, SBP, DBP, Heart Rate, Height, Weight, Abdominal Circumference, WBC, WBC Neutrophil, WBC Lymphocyte, Hemoglobin, Platelet, Glucose, Creatinine, Maximum Creatine Kinase Peek, Maximum Creatine Kinase MB, Troponin I, Troponin T, Total Cholesterol, Triglyceride, HDL, LDL, hs-CRP, NTproBNP, BNP, HbA1c, ARU, PRU, LVEF, RWMI, Discharge SBP, Discharge DBP, Discharge Heart Rate

*ECG, denotes electrocardiogram; MI, myocardial infarction; TIMI, thrombolysis in myocardial infarction; STEMI, ST-elevation myocardial infarction; NSTEMI, Non-ST elevation myocardial infarction; CAG, coronary angiogram; SBP, systolic blood pressure; DBP, diastolic blood pressure; WBC, white blood cells; HDL, high-density lipoprotein cholesterol; LDL, low-density lipoprotein cholesterol; hs-CRP, C-reactive protein; NTproBNP, N-terminal prohormone of brain natriuretic peptide; BNP, B-type natriuretic peptide; HbA1c, glycated hemoglobin; ARU, aspirin reactivity units; PRU, platelet reactivity units; LVEF, left ventricular ejection fraction; RWMI, regional wall motion index.

There are several missing values (e.g., heart rate, systolic blood pressure, diastolic blood pressure, white blood cells, etc.) in the dataset. Therefore, different approaches are used to preprocess the dataset before designing the prediction model, which mainly includes three parts: missing value imputation, feature selection, and data normalization.

#### 3.2.1 Missing value imputation

The collected dataset often contains several missing values, especially in the medical dataset. Firstly, we removed the features with more than 50% missing values in the dataset since those features may have a bad influence on the developed prediction models. Different types of methods have been used to handle the missing values which can be divided into two groups: statistical and ML-based techniques. Statistical techniques like mean and mode approaches are the simplest methods to impute the missing values in the data. The mean approach fills the missing values by the average value and the mode approach by the value that appears most often in the feature. The KNN is a representative supervised learning technique that is the most popular used ML method to impute the missing values based on the k nearest observed values (44). It has been shown that this imputation method is efficient in many types of research (45–47), and also includes tabular data (46). In this paper, the KNN-based imputation method is used to handle the missing values that use the k closest samples to determine the estimated missing value in the dataset, and k is set to 5.

#### 3.2.2 Wrapper-based feature selection

The feature selection method is used popularly in the medical field, and can be used for dimensionality reduction and the development of more efficient prediction models (48–50). In this paper, the RFE wrapper-based feature selection method is used with a 5-fold cross-validation approach to select the most important features for our target. Moreover, the number of selected features can be decided by the algorithm automatically in the wrapper-based feature selection method, where the RF algorithm is used as an estimator in the RFE wrapper-based feature selection method because it has shown better performance in many domains. This method is used to provide the same inputs to all prediction models and improve the final performance.

#### 3.2.3 Data normalization

ML algorithms compare the features in the data to find the patterns, there is a serious problem for the ML algorithms if the scale of the features in the data is severely different, especially for DL algorithms. Data normalization is a useful technique to normalize the scale of the features to a specific range such as between 0 and 1 or between −1 and 1, which can improve the performance as well as training stability of the ML and DL models (51). In this paper, the min-max normalization is used since it doesn't change the distribution of the original dataset. The calculation process of the method is shown in Equation (1).

(1) $$x_{scaled}=\;\frac{x-x_{min}}{x_{max}-\;x_{min}}$$

Where *x* stands for the set of original values, x_scaled the normalized value, x_min the minimum value in *x*, x_max the maximum value in *x*.

### 3.3 Cost-sensitive learning & threshold moving techniques

Cost-sensitive learning is the subfield of ML that considers the costs of misclassifications when dealing with classification problems. It is also a good solution for the class imbalance problem because it improves the generalization of the minority class by penalizing errors in that class and pushes the decision boundary away from these instances (52). It has been used popularly to address the class imbalance problem in different research (30, 33, 34, 53, 54). In cost-sensitive learning, the objective is to minimize the misclassification cost. The cost matrix of binary classification is shown in Table 2, where we use 1 for positive and 0 for negative.

**Table 2:** The cost matrix of binary classification.

	Predicted negative	Predicted positive
Actual negative	Cost (0, 0)	Cost (1, 0)
Actual positive	Cost (0, 1)	Cost (1, 1)

The instance cost of misclassification is measured by the Cost(i,j), which corresponds to the misclassification costs of classifying j into its predicted class i (55). The cost of the correct classification, Cost (0,0) and Cost (1,1) are zero. To estimate the cost value of the misclassification, the imbalance ratio (IR) as shown in Equation (2) is used popularly, which can be calculated as the quotient of the number of majority samples by the number of samples in the minority class. In addition, the misclassification cost value can also be considered a hyperparameter in the model. The *class_weight* is a parameter in Python language used to learn the cost-sensitive learning for most of the baseline classification algorithms.

(2) $$\mathrm{IR}=\frac{\mathrm{number}\;\mathrm{of}\;\mathrm{majority}\;\mathrm{samples}}{\mathrm{number}\;\mathrm{of}\;\mathrm{minority}\;\mathrm{samples}}$$

Many ML algorithms are designed to predict the probability of the class in terms of a default probability threshold of 0.5, which means that values equal to or exceeding the threshold are assigned to one class and all other values to another (31). However, the default threshold may lead to poor performance of the algorithms if there is a serious class imbalance issue in the dataset. The threshold moving technique (35) is used to handle the class imbalance problem which uses the original training data to train a model and then moves the decision probability threshold to predict the minority samples more accurately. Therefore, distinct threshold values are employed and then evaluated the label based on a selected evaluation matrix. The threshold that yields the best evaluation matrix will be used when predicting unseen data in the future.

### 3.4 Proposed method

DL methods have been applied to different types of data, such as image data, tabular data, text data, voice data, etc., and have shown adequate advantages in different domains recently. In this paper, a CSDNN-based method is proposed with a threshold moving technique to predict the mortality in out-of-hospital AMI patients with hypertension on imbalanced tabular data. To develop a more accurate DL-based model, we split the validation data (10% of the full data) from the training data, which is used to tune the hyperparameters and avoid the overfitting problem in the training process. Then we evaluate the performance of the proposed model with optimal hyperparameters on the test data (20%).

**Figure 2:**
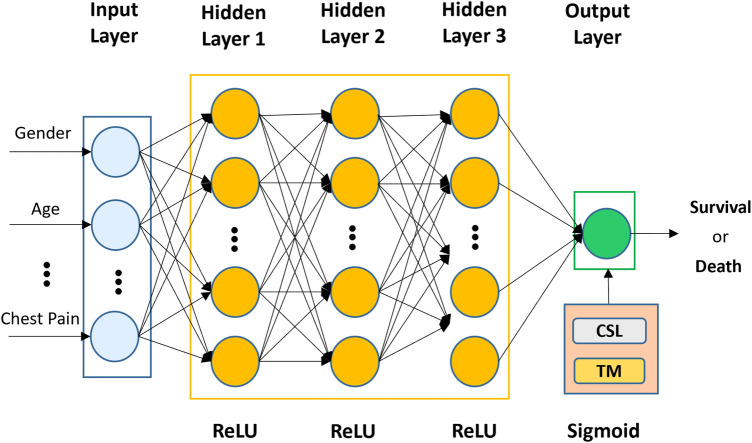
The architecture of the proposed CSDNN-based mortality prediction model with the threshold moving technique on imbalanced data.

The architecture of the proposed CSDNN-based mortality prediction model is shown in Figure 2, which mainly consists of an input layer, three hidden layers, and an output layer. In the first part, the selected features from the dataset (e.g., gender, age, chest pain, etc.) are used as input to the input layer and then propagated to the subsequent layers. In the second step, three hidden layers are used with 20, 20, and 15 neurons and are fully connected, where the optimal hidden neurons are obtained from the hyperparameter optimization method. In the output layer, the result is produced for given inputs. To overcome the class imbalance problem between healthy individuals (majority) and patients (minority), the cost-sensitive learning technique is applied to the proposed method with the optimal weight value, which gives a much higher *class_weight* value to the patient's records. Moreover, the threshold moving technique is used to improve the performance which moves the decision probability threshold to maximize the prediction performance of the patient's class when training the prediction model. To solve this binary classification problem, the binary cross entropy (56) is used as the loss function which compares each of the predicted probabilities to the actual class output and then calculates the score that penalizes the probabilities based on the distance between the predicted and the actual values. Additionally, to minimize the loss and to achieve more accurate outputs in the neural network training process, the backward propagation algorithm (57) is used to fine-tune the weights. The whole process of the neural network computation and the binary cross entropy can be expressed as Equations (3, 4).

(3) $$y=\varphi^{\left(4\right)}(w_{4}*(\varphi^{\left(3\right)}(w_{3}*\varphi^{\left(2\right)}(w_{2}*\varphi^{\left(1\right)}\;(w_{1}*x+b_{1})+b_{2})+b_{3})+b_{4})$$

(4) $$loss=-\frac{1}{N}\overset{N}{\underset{i=1}{\sum }}y_{i}\cdot \log(\;p(y_{i}))+(1-y_{i})\cdot \log(1-p(y_{i}))$$

where *x* represents the input units from the previous layer, $w_{i}$ and $b_{i}$ are the weight matrix and bias vector in each layer, respectively, $\varphi^{\left(i\right)}$ is the activation function, *N* is the number of samples, $p(y_{i})$ is the probability of a positive class, and $\left(1-p(y_{i})\right)$ is the probability of a negative class.

The activation function $\varphi^{\left(i\right)}$ is typically a nonlinear function and plays an important role in determining neuron activation. Without activation functions, the data would move through the network's nodes and layers using just linear functions, which are unable to recognize complicated patterns in the data. Several types of activation functions are used popularly, such as the rectified linear unit (ReLU), sigmoid, Tanh, etc (58). The ReLU is the most popular choice of activation function for hidden layers because it is easy to compute and does not make the problem of vanishing gradient. In this paper, the ReLU function is applied in all hidden layers because of its efficiency, and the sigmoid function is used in the output layer since our target feature is a binary-valued variable. The mathematical representations of the ReLU and sigmoid functions are shown in Equations (5, 6), where x denotes the input value.

(5) φ(x)=max{0,x}

(6) $$\varphi (x)=\;\frac{1}{1+\;e^{-x}}$$

In addition, the Adam optimizer, which is more efficient and can automatically reduce the learning rate, is used to optimize the weight with a learning rate of 0.01 (59). The batch size is given as 32, and the early stopping technique is applied with the patience of 30 to avoid overfitting and improve the speed of model development (60).

### 3.5 Compared methods

Some commonly used ML and ensemble methods such as SVM (61), LR (62), DT (63), RF (64), AdaBoost (65), and XGBoost (66), have shown better performance in different domains (5, 14, 15, 41, 54, 67). Therefore, we compared these models with the proposed CSDNN-based method to estimate the performance of the original imbalanced data with and without feature selection, cost-sensitive learning, and threshold moving technique. In addition, several classic data sampling-based ensemble methods such as balanced bagging (37), balanced RF (38), EasyEnsemble (68), and RUSBoost classifiers (69) are also applied with the feature selection and threshold moving technique to check the robustness of the proposed method. A brief description of these methods is as follows.

SVM (61) is a powerful method that seeks to identify an optimal decision boundary called hyperplane with maximum margin to classify the data points of both classes distinctly. Different kernel functions can be used to solve nonlinear problems. In this study, linear support vector classification (LinearSVC) (70) is used as an alternative to the traditional SVM with kernel functions due to its flexibility and speed for large datasets. LR (62) is a useful analysis method to solve binary classification problems by using a sigmoid function to squash the value range between 0 and 1. DT (63) is one of the most efficient ML algorithms and performs well on large datasets. It aims to predict the variable's values by learning from simple decisions. Several ensemble ML algorithms are also applied in this experiment. RF (64) is the famous ensemble method that constructs numerous decision trees by using the DT algorithm as a base estimator with a bagging approach at training time and finally outputs the result that most trees select. AdaBoost (65) is the typical boosting ensemble method that combines multiple weak estimators to generate the strong estimator by adaptively assigning the higher weight to misclassified instances and has shown its effectiveness in producing a more accurate model. XGBoost (66), the gradient-boosting framework, is used to build the decision tree-based ensemble. It has shown good performance and computational speed to handle classification and regression problems.

Several classic data sampling-based ensemble methods are also used in the experimental analysis. The balanced bagging method (37) uses all of the minority samples by undersampling the majority classes to improve the original bagging algorithm with skewed class distributions. Balanced RF classifier (38) takes the bootstrap samples from the minority class for each iteration of RF and then randomly undersamples the same number of replacement samples from the majority class to balance the dataset. EasyEnsemble classifier (68) is the ensemble of AdaBoost estimators which are trained on different balanced bootstrap samples by using the random undersampling technique to select the subset from the majority class and all instances from the minority class. RUSBoost classifier (61) randomly undersamples the dataset at each iteration to balance the class distribution while the AdaBoost algorithm is used to improve the performance using the balanced data.

### 3.6 Hyperparameter optimization

A range of hyperparameter optimization methods is used frequently to customize and generate a more accurate prediction model. For example, random search (71) and grid search (72) are the simplest and most popular methods for hyperparameter optimization. In random search, search space is the bounded set of parameters with randomly chosen values, whereas the grid search method consists of a set of hyperparameter values and evaluates every position along the grid. The key difference between these methods is that only a few values are tested and chosen randomly in the random search. The performance of these methods is similar in small datasets, whereas the random search method is faster than the grid search method in large datasets. Several other references (15, 17, 69, 73) have been consulted in determining the parameters that may have a significant impact on the results of ML-based methods. In this paper, the random search with stratified 5-fold cross-validation is used to set the parameters of our proposed method and other compared methods because of the efficiency. The parameters and ranges of each algorithm were selected based on many references and our pre-experiment, as shown in Table 3. Moreover, to obtain the best value from all possible values of the *class_weight* parameter in our proposed method, the grid search with stratified 5-fold cross-validation is applied.

**Table 3:** Hyperparameter optimization of all machine learning algorithms with random search approach.

Algorithm	Parameter	Range
LR	Penalty	{‘l1’, ‘l2’, ‘elasticnet’, ‘none’}
	Solver	{‘newton-cg’, ‘lbfgs’, ‘liblinear’, ‘sag’, ‘saga’}
	C	{1e-5, 1e-4, 1e-3, 1e-2, 1e-1, 1, 10, 100, 150, 200, 250, 300, 350, 400, 450, 500}
DT	Criterion	{‘gini’, ‘entropy’}
RF	Number of estimators	{100, 150, 200, 250, 300, 350, 400, 450, 500, 600, 700, 800, 900, 1000,1100, 1200, 1300, 1400, 1500}
	Criterion	{‘gini’, ‘entropy’}
XGBoost	Number of estimators	{100, 150, 200, 250, 300, 350, 400, 450, 500, 600, 700, 800, 900, 1000,1100, 1200, 1300, 1400, 1500}
	eta (learning rate)	{0.001, 0.005, 0.01, 0.05, 0.1, 0.15, 0.2, 0.25, 0.3}
	Maximum depth	(3, 10)
	Gamma	{0, 0.1, 0.2, 0.3, 0.4, 0.5)
AdaBoost	Number of estimators	{100, 150, 200, 250, 300, 350, 400, 450, 500, 600, 700, 800, 900, 1000,1100, 1200, 1300, 1400, 1500}
	learning rate	{0.001, 0.005, 0.01, 0.05, 0.1, 0.15, 0.2, 0.25, 0.3}
Balanced Bagging	Number of estimators	{100, 150, 200, 250, 300, 350, 400, 450, 500, 600, 700, 800, 900, 1000,1100, 1200, 1300, 1400, 1500}
Balanced RF	Number of estimators	{100, 150, 200, 250, 300, 350, 400, 450, 500, 600, 700, 800, 900, 1000,1100, 1200, 1300, 1400, 1500}
EasyEnsemble	Number of estimators	{100, 150, 200, 250, 300, 350, 400, 450, 500, 600, 700, 800, 900, 1000,1100, 1200, 1300, 1400, 1500}
RUSBoost	Number of estimators	{100, 150, 200, 250, 300, 350, 400, 450, 500, 600, 700, 800, 900, 1000,1100, 1200, 1300, 1400, 1500}
DNN	Number of neurons in each layer	{2, 3, 5, 7, 10, 13, 15, 17, 19, 20, 25, 27, 30, 35, 40, 50, 55}
	Learning rate	{0.0001, 0.001, 0.005, 0.01, 0.05, 0.1}

### 3.7 Statistical analysis and implementation environments

To analyze the categorical (i.e., gender, chest pain, etc.) and continuous (i.e., age, height, weight, etc.) variables in experimental data, we apply the Chi-square test (74) and independent *t*-test (75), respectively. In categorical variables, frequency and proportion are expressed, while continuous variables are expressed as mean value and standard deviation. Moreover, the significance level of *p* < 0.05 for statistical significance is used in this experiment.

We implemented all experiments on a Microsoft Windows server with Intel Xeon CPU E5-2696 v5 @ 4.40 GHz, 512GB random access memory (RAM), and NVIDIA GeForce GTX 1080 24 GB, and used IBM SPSS Statistics 23 for statistical analysis, and Python language (Version 3.6) in Jupyter Notebook (76) with scikit-learn (77), Tensorflow (78), Keras (79), imbalanced-learn (80) packages, and xgboost library (81), for data preprocessing and designing the prediction models.

### 3.8 Performance evaluation measures

Generally, standard performance measures such as accuracy, recall, precision, etc. are widely adopted for balanced datasets to estimate the results of the predictive models. However, the use of common metrics can mislead the results in a dataset with a skewed distribution. Especially in the medical domain, diagnosing the patient from general people for timely treatment can be seriously affected, and die in the worst situations. In addition, misdiagnosis of general people will cause a lot of unnecessary treatment costs and waste of medical resources. The performance of our proposed mortality prediction model will be evaluated by the balanced accuracy, area under the receiver operating characteristic curve (AUC), macro-averaged precision, recall, F1-score, and geometric mean (g-mean), where the macro-average gives equal weight to each class and compute the metric individually and then take the average. The mathematical expressions of the performance measures are shown in Equation (10–14), where true positive, false positive, true negative, and false negative in the confusion matrix are expressed as TP, FP, TN, and FN, respectively.

(7) $$\mathrm{Precision}=\frac{\mathrm{TP}}{\mathrm{TP}+\mathrm{FP}}$$

(8) $$\mathrm{Sensitivity}=\mathrm{Recall}=\frac{\mathrm{TP}}{\mathrm{TP}+\mathrm{FN}}$$

(9) $$\mathrm{Specificity}=\frac{\mathrm{TN}}{\mathrm{TN}+\mathrm{FP}}$$

(10) $$\mathrm{Precisio}n_{\mathrm{macro}}=\frac{\mathrm{precisio}n_{1}+\mathrm{precisio}n_{2}}{2}$$

(11) $$\mathrm{Recal}l_{\mathrm{macro}}=\frac{\mathrm{Recal}l_{1}+\mathrm{Recal}l_{2}}{2}$$

(12) $$\mathrm{Balanced}\;\mathrm{Accuracy}=\frac{\mathrm{sensitivity}+\mathrm{specificity}}{2}=\frac{\frac{\mathrm{TP}}{\mathrm{TP}+\mathrm{FN}}+\frac{\mathrm{TN}}{\mathrm{TN}+\mathrm{FP}}}{2}$$

(13) $$F1-\mathrm{scor}e_{\mathrm{macro}}=2*\frac{\mathrm{Precisio}n_{\mathrm{macro}}*\mathrm{Recal}l_{\mathrm{macro}}}{\mathrm{Precisio}n_{\mathrm{macro}}+\mathrm{Recal}l_{\mathrm{macro}}}$$

(14) $$G-\mathrm{mea}n_{\mathrm{macro}}=\sqrt{\mathrm{Recal}l_{\mathrm{macro}}*\mathrm{Specificit}y_{\mathrm{macro}}}=\sqrt{\frac{\mathrm{Recal}l_{1}+\mathrm{Recal}l_{2}}{2}*\frac{\mathrm{Specificit}y_{1}+\mathrm{Specificit}y_{2}}{2}}$$

## 4 Results and discussion

### 4.1 Baseline characteristics

From the raw dataset, out-of-hospital AMI patients' data with hypertension (*N* = 5,602) was extracted as the experimental dataset which contained the survived patients of 5,402 (96.43%) and deceased patients 200 (3.57%) with 2-year follow-ups. Table 4 summarized the baseline characteristics of demographic information, clinical findings, medical history, and laboratory findings between the survived and deceased groups, and variables that were statistically significant between the two groups were boldfaced. The results showed that males were more likely to have AMI with hypertension than females. The mean age of the patients was 66.19 ± 11.68 years, and there was a difference of about 9 years between the survived group (65.87 ± 11.65) and the deceased group (74.69 ± 9.03) and was statistically significant (*p* ≤ 0.001***). In addition, the variables gender, age, weight, chest pain (typical), dyspnea (yes), heart rate, Killip class, current smoker (yes), LVEF, RWMI, history of diabetes mellitus, previous angina pectoris (yes), previous heart failure (yes), previous cerebrovascular disease (yes), neutrophil, lymphocyte, hemoglobin, glucose, creatinine, total cholesterol, triglyceride, hs-CRP, NTproBNP, BNP, and PRU, were statistically significant with *p*-value ≤ 0.001, as well as height ≤ 0.01, previous myocardial infarction (yes) ≤ 0.01, LDL ≤ 0.01, previous chest pain (yes) ≤ 0.05, use of CAG ≤ 0.05, HbA1c ≤ 0.05, ARU ≤ 0.05, respectively. On the other hand, abdominal circumference, SBP, DBP, ECG (yes), ST_change on ECG (yes), symptoms of MI (yes), MI ECG change (yes), use of thrombolysis, use of Echocardiogram, history of dyslipidemia (yes), family history of heart disease (yes), family history of early age ischemic heart disease (yes), WBC, platelet, maximum creatine kinase peek, maximum creatine kinase MB, troponin I, troponin T, and HDL were least significant with *p*-value > 0.05.

**Table 4:** The baseline characteristics of survived and deceased groups.

Variable	All (*N* = 5,602)	Survival (*N* = 5,402)	Death (*N* = 200)	*p*-value
Demographic characteristics				
Gender	5,602	5,402 (96.43)	200 (3.57)	**≤0**.001***
Men	3,778 (67.4)	3,669 (67.9)	109 (54.5)	
Women	1,824 (32.6)	1,733 (32.1)	91 (45.5)	
Age (years)	66.19 ± 11.68	65.87 ± 11.65	74.69 ± 9.03	**≤0**.001***
Height (cm)	162.95 ± 11.26	163.03 ± 11.33	160.77 ± 8.99	**≤0**.01**
Weight (kg)	65.29 ± 12.42	65.47 ± 12.41	60.304 ± 11.61	**≤0**.001***
Abdominal circumference (cm)	88.76 ± 9.03	88.8 ± 9.01	87.88 ± 9.49	0.385
Clinical findings				
Chest pain (typical)	4,856 (86.7)	4,773 (87.2)	143 (71.5)	**≤0**.001***
Dyspnea (yes)	1,342 (24.0)	1,269 (23.5)	73 (36.5)	**≤0**.001***
Previous chest pain (yes)	1,492 (26.6)	1,454 (26.9)	38 (19.0)	**≤0**.05*
SBP (mmHg)	133.64 ± 29.12	133.72 ± 29.14	131.45 ± 28.58	0.28
DBP (mmHg)	79.83 ± 17.32	79.86 ± 17.35	78.93 ± 16.66	0.456
Heart rate (bpm)	78.52 ± 19.06	78.3 ± 18.95	84.62 ± 20.97	**≤0**.001***
Killip class	5,602	5,402 (96.43)	200 (3.57)	**≤0**.001***
Ⅰ	4,452 (79.5)	4,344 (80.4)	108 (54.0)	
Ⅱ	501 (8.9)	466 (8.6)	35 (17.5)	
Ⅲ	418 (7.5)	368 (6.8)	50 (25.0)	
Ⅳ	231 (4.1)	224 (4.1)	7 (3.5)	
ECG (Yes)	5,589 (99.8)	5,390 (99.8)	199 (99.5)	0.423
ST_change on ECG (yes)	4,319 (77.1)	4,165 (77.1)	154 (77.0)	0.973
Current smoker (yes)	1,698 (30.3)	1,672 (31.0)	26 (13.0)	**≤0**.001***
Symptoms of MI (yes)	5,490 (98.0)	5,296 (98.0)	194 (97.0)	0.303
MI ECG change (yes)	3,745 (66.9)	3,608 (66.8)	137 (68.5)	0.614
Use of Thrombolysis	46 (0.8)	45 (0.8)	1 (0.5)	0.608
Use of CAG	5,545 (99.0)	5,350 (99.0)	195 (97.5)	**≤0**.05*
Use of echocardiogram	5,437 (97.1)	5,244 (97.1)	193 (96.5)	0.637
LVEF	52.84 ± 10.95	53.09 ± 10.82	45.93 ± 12.03	**≤0**.001***
RWMI	1.4 ± 0.38	1.39 ± 0.38	1.53 ± 0.43	**≤0**.001***
Medical history				
History of diabetes mellitus (yes)	2,036 (36.3)	1,930 (35.7)	106 (53.0)	**≤0**.001***
History of dyslipidemia (yes)	821 (14.7)	801 (14.8)	20 (10.0)	0.058
Previous myocardial infarction (yes)	478 (8.5)	449 (8.3)	29 (14.5)	**≤0**.01**
Previous angina pectoris (yes)	684 (12.2)	645 (11.9)	39 (19.5)	**≤0**.001***
Previous heart failure (yes)	99 (1.8)	87 (1.6)	12 (6.0)	**≤0**.001***
Previous cerebrovascular disease (yes)	538 (9.6)	503 (9.3)	35 (17.5)	**≤0**.001***
Family history of heart disease (yes)	332 (5.9)	324 (6.0)	8 (4.0)	0.234
Family history of early AGE ischemic heart disease (yes)	38 (11.4)	37 (11.4)	1 (12.5)	0.924
Laboratory Findings				
WBC (10^3^/mL)	10.05 ± 3.74	10.06 ± 3.74	9.68 ± 3.68	0.155
Neutrophil (%)	66.41 ± 14.6	66.27 ± 14.58	70.29 ± 14.6	**≤0**.001***
Lymphocyte (%)	24.59 ± 12.48	24.72 ± 12.44	21.09 ± 12.90	**≤0**.001***
Hemoglobin (g/dl)	13.53 ± 2.1	13.60 ± 2.06	11.7 ± 2.37	**≤0**.001***
Platelet (mm)	231.99 ± 66.81	232.22 ± 65.8	225.76 ± 89.86	0.179
Glucose (mg/dl)	170.55 ± 78.59	169.81 ± 78.06	190.52 ± 89.61	**≤0**.001***
Creatinine (mg/dl)	1.2 ± 1.37	1.16 ± 1.3	2.27 ± 2.42	**≤0**.001***
Maximum creatine kinase peek (mg/dl)	835.71 ± 1,434.54	831.74 ± 1,427.16	937.73 ± 1,613.83	0.334
Maximum creatine kinase MB (mg/dl)	96.88 ± 160.07	97.54 ± 161.47	78.96 ± 114.82	0.11
Troponin I (mg/dl)	41.2 ± 104.95	41.07 ± 105.29	44.33 ± 96.01	0.68
Troponin T (mg/dl)	28.71 ± 701.81	29.18 ± 708.73	5.05 ± 5.62	0.892
Total cholesterol (mg/dl)	172.56 ± 43.36	173.04 ± 43.34	159.68 ± 41.94	**≤0**.001***
Triglyceride (mg/dl)	133.55 ± 111.22	134.69 ± 112.59	102.88 ± 56.53	**≤0**.001***
HDL (mg/dl)	43.01 ± 12.83	43.07 ± 12.74	41.6 ± 14.88	0.124
LDL (mg/dl)	106.04 ± 36.68	106.35 ± 36.66	97.72 ± 36.09	**≤0**.01**
hs-CRP (mg/dl)	1.43 ± 6.33	1.27 ± 4.53	6.4 ± 25.27	**≤0**.001***
NTproBNP (mg/dl)	2,518.07 ± 6,836.86	2,221.3 ± 6,239.58	9,333.16 ± 13,496.1	**≤0**.001***
BNP (mg/dl)	348.56 ± 789.73	327.21 ± 755.39	1,360.11 ± 1,492.84	**≤0**.001***
HbA1c (%)	6.54 ± 1.42	6.53 ± 1.41	6.81 ± 1.57	**≤0**.05*
ARU (units)	458.9 ± 71.05	457.94 ± 71.07	478.4 ± 68.45	**≤0**.05*
PRU (units)	207.56 ± 110.75	204.87 ± 110.23	270.59 ± 104.68	**≤0**.001***

* SBP, denotes systolic blood pressure; DBP, diastolic blood pressure; ECG, electrocardiogram; MI, myocardial infarction; CAG, coronary angiogram; LVEF, left ventricular ejection fraction; RWMI, regional wall motion index; WBC, white blood cells; HDL, high-density lipoprotein cholesterol; LDL, low-density Lipoprotein cholesterol; hs-CRP, C-reactive protein; NTproBNP, N-terminal prohormone of brain natriuretic peptide; BNP, B-type natriuretic peptide; HbA1c, glycated hemoglobin; ARU, aspirin reactivity units, PRU, platelet reactivity units. The *p*-value with *, **, and ***denote that each variable is statistically significant as *p*-value ≤ 0.05, <0.01, and <0.001 in survived and deceased groups, which means there are less than 5%, 1%, and 0.1% as chances of being incorrect, respectively.

### 4.2 Results of prediction models

In this part, we examined the performance of the proposed CSDNN-based model as well as other famous ML-based models such as SVM, LR, DT, RF, AdaBoost, XGBoost, and classic data sampling-based ensemble models such as balanced bagging, balanced RF, EasyEnsemble, and RUSBoost for the mortality prediction of out of hospital AMI patients with hypertension. The performance was evaluated using balanced accuracy, AUC, macro-averaged precision, recall, F1-score, and g-mean. Tables 5–7 showed the performance comparison results of the proposed model and other prediction models on the original imbalanced data with and without feature selection, cost-sensitive learning, and threshold moving technique. The boldface expresses the best performance among compared models.

**Table 5:** Performance comparison of the proposed and machine learning-based prediction models without applying feature selection.

Cost-sensitive learning	Threshold moving	Model	Balanced accuracy	Precision	Recall	F1-score	G_mean	AUC	Threshold
None	None	LR	0.5128	**0** **.** **983**	0.5128	0.5163	0.5128	0.5128	–
		SVM	0.5128	**0**.**983**	0.5128	0.5163	0.5128	0.5128	–
		DT	**0**.**5935**	0.5455	**0**.**5935**	0.557	**0**.**5935**	**0**.**5935**	–
		RF	0.5	0.4826	0.5	0.4911	0.5	0.5	–
		XGBoost	0.4982	0.4825	0.4982	0.4902	0.4982	0.4982	–
		AdaBoost	0.5457	0.6092	0.5457	0.562	0.5457	0.5457	–
		DNN	0.5609	0.693	0.5609	**0**.**5887**	0.5609	0.5609	–
With cost–sensitive learning [Class weight = (0:1, 1:27.01)]	None	LR	0.7221	0.5446	0.7221	0.5325	0.7221	0.7221	–
		SVM	**0**.**7317**	0.5454	**0**.**7317**	0.5317	**0**.**7317**	**0**.**7317**	–
		DT	0.5095	0.51	0.5095	0.5097	0.5095	0.5095	–
		RF	0.5	0.4826	0.5	0.4911	0.5	0.5	–
		XGBoost	0.6866	0.5485	0.6866	0.5534	0.6866	0.6866	–
		AdaBoost	0.652	**0**.**5495**	0.652	**0**.**5608**	0.652	0.652	–
		CSDNN	**0**.**7317**	0.5454	**0**.**7317**	0.5317	**0**.**7317**	**0**.**7317**	–
	With threshold moving	LR	0.6354	0.6028	0.6354	**0**.**6163**	0.6354	0.6354	0.87
		SVM	–	–	–	–	–	–	–
		DT	0.5095	0.51	0.5095	0.5097	0.5095	0.5095	0
		RF	0.6631	0.5634	0.6631	0.5824	0.6631	0.6631	0.092
		XGBoost	0.6431	0.5893	0.6431	0.6082	0.6431	0.6431	0.700
		AdaBoost	0.5915	**0**.**6108**	0.5915	0.6	0.5915	0.5915	0.504
		CSDNN	**0**.**7354**	0.5809	**0**.**7354**	0.6074	**0**.**7354**	**0**.**7354**	0.681

**Table 6:** Performance comparison of the proposed and machine learning-based prediction models with feature selection.

Cost-sensitive learning	Threshold moving	Model	Balanced accuracy	Precision	Recall	F1-score	G_mean	AUC	Threshold
With cost-sensitive learning [Class weight = (0:1, 1:27.01)]	None	LR	0.7167	0.5291	0.7167	0.4089	0.7167	0.7167	–
		SVM	0.7164	0.5389	0.7164	0.5126	0.7164	0.7164	–
		DT	0.5072	0.5067	0.5072	0.5069	0.5072	0.5072	–
		RF	0.5	0.4826	0.5	0.4911	0.5	0.5	–
		XGBoost	0.6802	0.5439	0.6802	0.5437	0.6802	0.6802	–
		AdaBoost	0.7409	0.5503	0.7409	0.5442	0.7409	0.7409	–
		CSDNN	**0**.**7415**	**0**.**5562**	**0**.**7415**	**0**.**5603**	**0**.**7415**	**0**.**7415**	–
	With threshold moving	LR	0.674	0.565	0.674	0.5846	0.674	0.674	0.504
		SVM	–	–	–	–	–	–	–
		DT	0.5072	0.5067	0.5072	0.5069	0.5072	0.5072	0
		RF	0.715	0.557	0.715	0.567	0.715	0.715	0.071
		XGBoost	0.6125	**0**.**6002**	0.6125	**0**.**6059**	0.6125	0.6125	0.752
		AdaBoost	0.6877	0.5577	0.6877	0.572	0.6877	0.6877	0.505
		CSDNN	**0**.**7634**	0.559	**0**.**7634**	0.5623	**0**.**7634**	**0**.**7634**	0.489

**Table 7:** Performance comparison of the proposed and classic data sampling-based ensemble prediction models with feature selection.

Cost-Sensitive Learning	Threshold moving	Model	Balanced accuracy	Precision	Recall	F1-score	G_mean	AUC	Threshold
None	None	Balanced Bagging (36)	0.7231	0.5498	0.7231	0.548	0.7231	0.7231	–
		Balanced RF (37)	0.7112	0.5348	0.7112	0.4947	0.7112	0.7112	–
		EasyEnsemble (38)	0.7412	0.5367	0.7412	0.4817	0.7412	0.7412	–
		RUSBoost (68)	0.6152	0.5212	0.6152	0.4886	0.6152	0.6152	–
With cost-sensitive learning [Class weight = (0:1, 1:27.01)]		CSDNN	**0**.**7415**	**0**.**5562**	**0**.**7415**	**0**.**5603**	**0**.**7415**	**0**.**7415**	–
None	With threshold moving	Balanced Bagging (36)	0.7246	0.5573	0.7246	0.5661	0.7246	0.7246	0.534
		Balanced RF (37)	0.6635	0.5641	0.6635	0.5834	0.6635	0.6635	0.685
		Easy Ensemble (38)	0.5837	**0**.**6605**	0.5837	**0**.**6083**	0.5837	0.5837	0.548
		RUSBoost (68)	0.5484	0.5484	0.5484	0.5484	0.5484	0.5484	0.514
With cost-sensitive learning [Class weight = (0:1, 1:27.01)]		CSDNN	**0**.**7634**	0.559	**0**.**7634**	0.5623	**0**.**7634**	**0**.**7634**	0.489

A total of 63 independent features were used to predict the mortality from the original imbalanced data. Table 5 showed the results of balanced accuracy, AUC, macro-averaged precision, recall, F1-score, and g-mean of the proposed model and other state-of-the-art ML-based models without applying the feature selection method. It can be divided into three different cases, (1) without applying cost-sensitive learning and threshold moving technique, (2) using cost-sensitive learning but without moving threshold, and (3) using both cost-sensitive learning and threshold moving technique. Moreover, the *class_weight* value of the cost-sensitive learning method applied in each algorithm was {0:1, 1:27.01}, which meant the class weight of the minority class was set up to 27.01 calculated by the IR. As shown in Table 5, it was evident that the issue of class imbalance affected every model. In the first case, the DT model got comparatively higher performance among all other models without using the cost-sensitive learning and threshold moving technique to solve the class imbalance problem. In the second case, the CSDNN model and SVM outperformed the other five models with cost-sensitive learning and without threshold moving techniques. Moreover, the CSDNN model showed the highest balanced accuracy of 0.7354, macro-averaged recall 0.7354, g-mean 0.7354, and AUC 0.7354, in the third case using both cost-sensitive learning and threshold moving technique. The *class_weight* value of {0:1, 1:27.01} was used in the proposed model and other ML-based prediction models with the cost-sensitive learning method. The default probability threshold of all classifiers is 0.5. However, the SVM prediction model with the threshold moving technique could not be applied since the LinearSVC classifier cannot predict the class probability of the samples.

After applying the RFE wrapper-based feature selection with a 5-fold cross-validation approach on the extracted experimental data, 27 optimal features were selected to predict the mortality in out-of-hospital AMI patients with hypertension. The optimal feature set consisted of age, heart rate, height, weight, abdominal circumference, WBC, neutrophil, lymphocyte, hemoglobin, platelet, glucose, creatinine, maximum creatine kinase peek, maximum creatine kinase MB, troponin I, troponin T, total cholesterol, HDL, LDL, hs-CRP, NTproBNP, BNP, ARU, PRU, LVEF, RWMI, and discharge heart rate. In Table 6, the performances of the proposed CSDNN-based model and other compared models with the optimal features were shown in two different cases. For instance, (1) using cost-sensitive learning but without moving threshold, (2) using both cost-sensitive learning and threshold moving techniques. The results demonstrated that the proposed model achieved the highest performance in both cases. The *class_weight* value of {0:1, 1:27.01} was also used for the cost-sensitive learning.

We also compared the performance of the proposed and classic data sampling-based ensemble prediction models. The performance comparison results of the proposed CSDNN model and classic data sampling-based ensemble models were shown in Table 7, which also included two cases, (1) without threshold moving, and (2) with threshold moving. The results indicated that the proposed CSDNN model obtained better performance than all data sampling-based ensemble prediction models in both cases.

**Figure 3:**
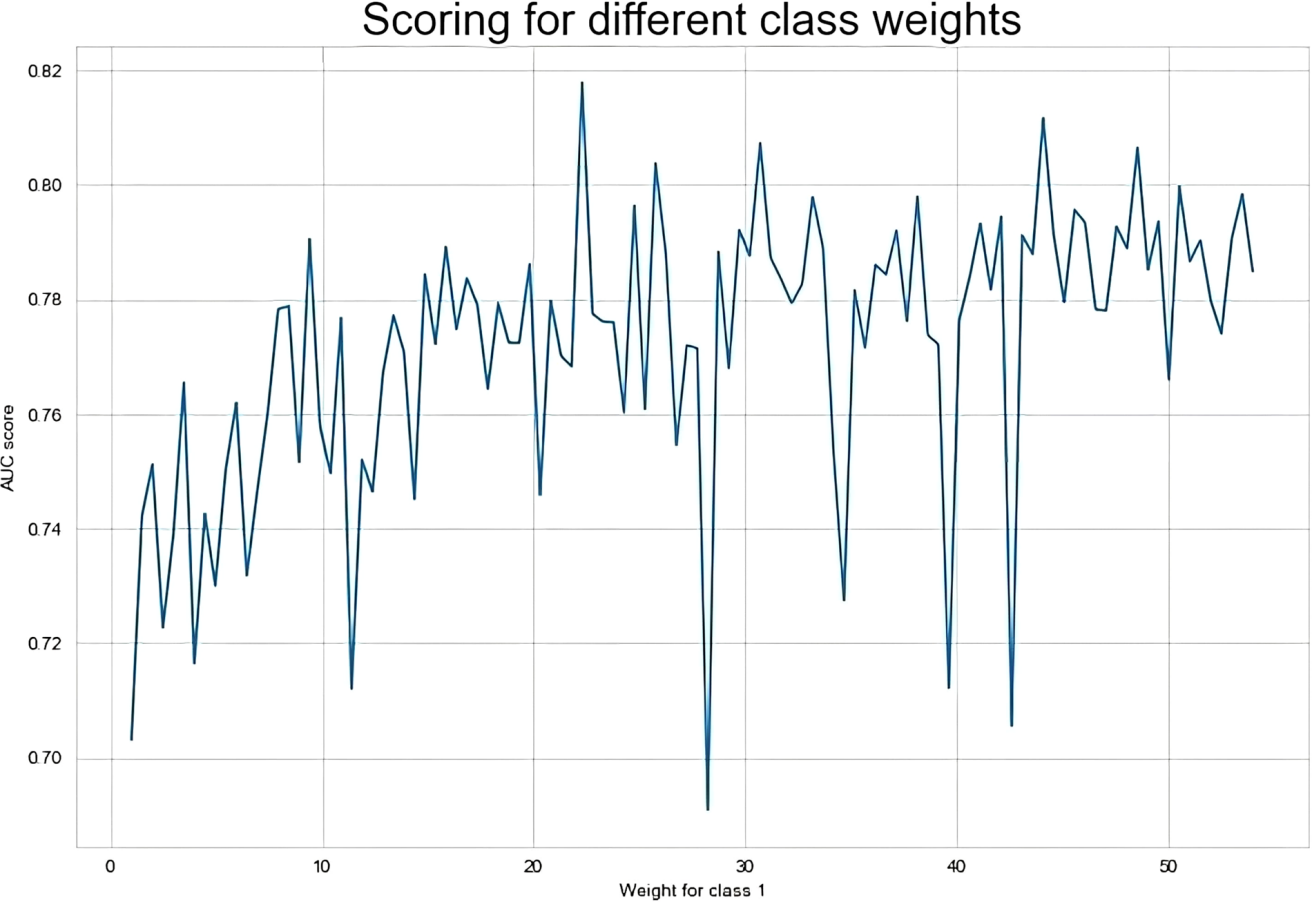
Result of the AUC score over different class weight values in the proposed model.

To develop a more accurate prediction model and search for the best value of the class_weight parameter, we applied the grid-search with 3-fold cross-validation for our proposed CSDNN method to obtain the best AUC score. The result of the AUC score for different class_weight values was shown in Figure 3, which demonstrated that the optimal class_weight value was {0:1, 1:22.3} for the minority class. To clearly understand the proposed method, Table 8 compared the performance difference of the proposed model with and without feature selection, cost-sensitive learning, and threshold moving technique. There were two kinds of cases of the threshold moving technique, with the default class_weight value of {0:1, 1:27.01} or the optimal class_weight value of {0:1, 1:22.3} for the minority class, respectively. The results showed that after we applied the optimal class_weight value as {0:1, 1:22.3} to our dataset, the performance of our proposed CSDNN model was increased with the balanced accuracy of 0.7667, macro-averaged precision 0.5613, recall 0.7667, F1-score 0.5675, g-mean 0.7667, and AUC 0.7667.

**Table 8:** Performance evaluation of the proposed models.

Feature selection	Cost-sensitive learning	Class_ weight	Threshold moving	Model	Balanced accuracy	Precision	Recall	F1-score	G_mean	AUC
None	None	None	None	DNN	0.5609	**0** **.** **693**	0.5609	0.5887	0.5609	0.5609
	With cost-sensitive learning	{0:1, 1:27.01}	None	CSDNN	0.7317	0.5454	0.7317	0.5317	0.7317	0.7317
		{0:1, 1:27.01}	With threshold moving	CSDNN	0.7354	0.5809	0.7354	**0**.**6074**	0.7354	0.7354
With feature selection		{0:1, 1:27.01}	None	CSDNN	0.7415	0.5562	0.7415	0.5603	0.7415	0.7415
		{0:1, 1:27.01}	With threshold moving	CSDNN	0.7634	0.559	0.7634	0.5623	0.7634	0.7634
		{0:1, 1:22.3}		CSDNN	**0**.**7667**	0.5613	**0**.**7667**	0.5675	**0**.**7667**	**0**.**7667**

**Figure 4:**
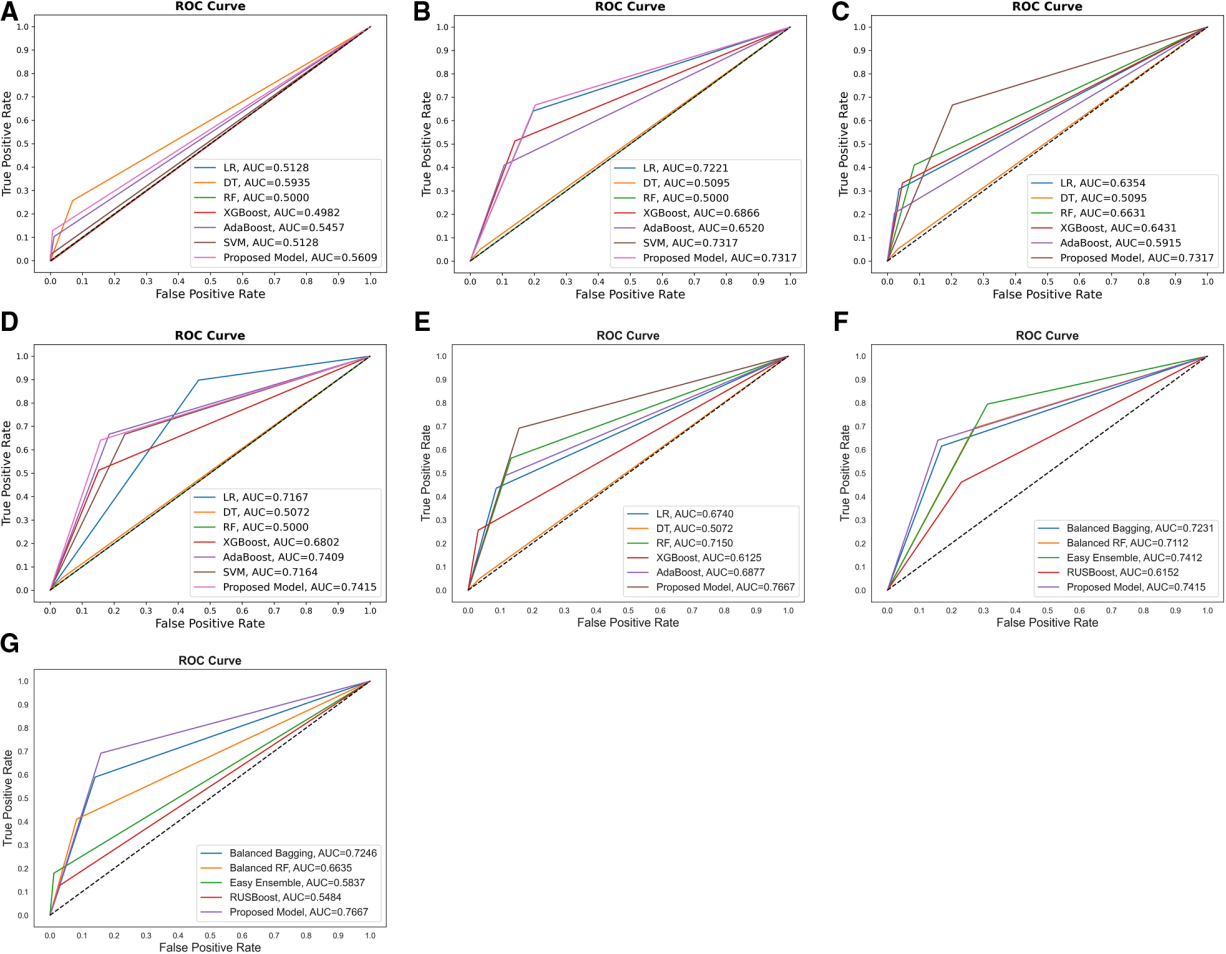
Comparison of the ROC curves of (**A**) proposed model and other ML models without feature selection, cost-sensitive learning, and threshold moving; (**B**) proposed model and others with cost-sensitive learning; (**C**) proposed model and others with cost-sensitive learning and threshold moving; (**D**) proposed model and others with feature selection and cost-sensitive learning; (**E**) proposed model and classic data sampling-based ensemble models with feature selection, cost-sensitive learning, and threshold moving; (**F**) proposed model and classic data sampling-based ensemble models with feature selection but without threshold moving; (**G**) proposed model and classic data sampling-based ensemble models with feature selection and threshold moving.

**Figure 5:**
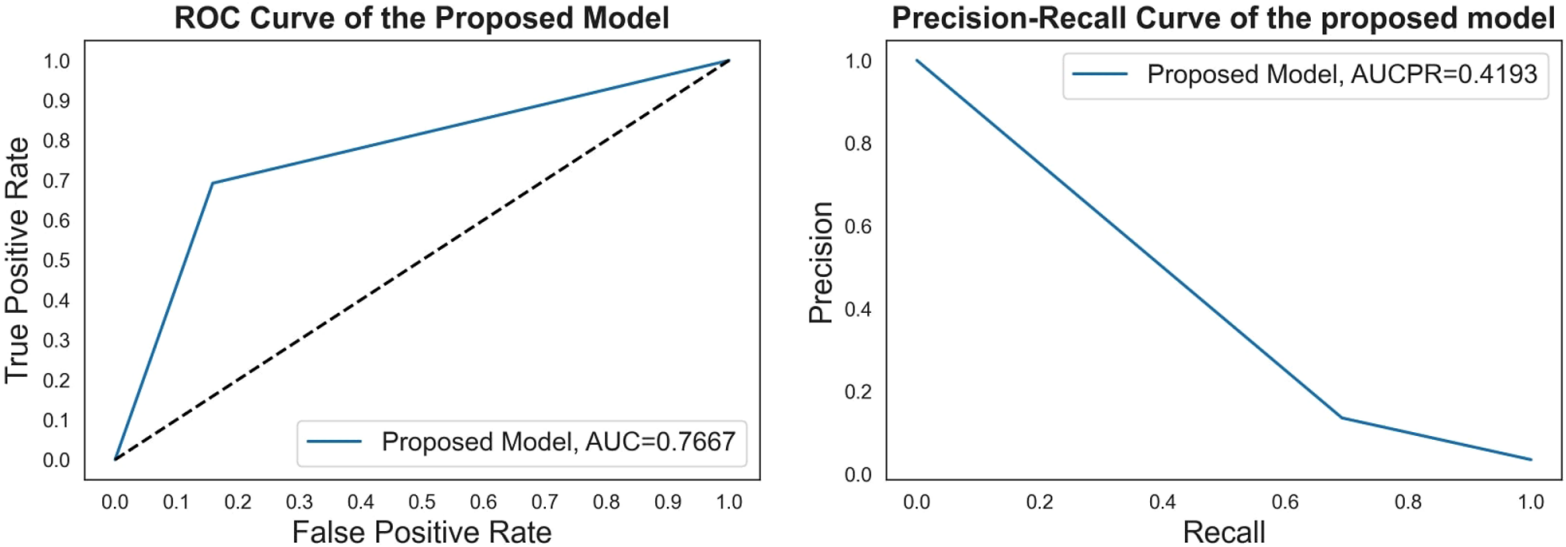
The ROC curve and precision-recall curve of the proposed model with feature selection, cost-sensitive learning, and threshold moving.

Additionally, the performance comparison of ROC curves on the proposed CSDNN model, state-of-the-art ML models, and classic data sampling-based ensemble models were also shown in Figure 4. Figures 4A–C showed the ROC curve comparisons between the CSDNN model and state-of-the-art ML classifiers on the three cases without applying the feature selection method on the original dataset, and Figures 4E–G showed the ROC curve comparisons between the CSDNN model, ML classifiers, and classic data sampling-based ensemble models on prescribed four cases with optimal features. As a result, ROC curves comparison in Figure 4A showed that the DT model obtained the best AUC of 0.5935 without feature selection, cost-sensitive learning, and threshold moving technique. In Figure 4B, the proposed model and SVM model exhibited the highest AUC of 0.7317 than other ML models, and the proposed model got a better AUC of 0.7354 than the other four models using the threshold moving technique as shown in Figure 4C. Figures 4D–G showed the ROC curves comparison of the proposed model with other models using the optimal features. In Figures 4D, F the proposed CSDNN model showed the best AUC of 0.7415 without moving the threshold, which was higher than the other models. Figures 4E, G demonstrated that the proposed model achieved the best AUC of 0.7667 with the optimal class_weight value. Additionally, Figure 5 demonstrated the ROC curve and precision-recall curve of the proposed model with the highest performance with feature selection, cost-sensitive learning, and threshold moving.

**Figure 6:**
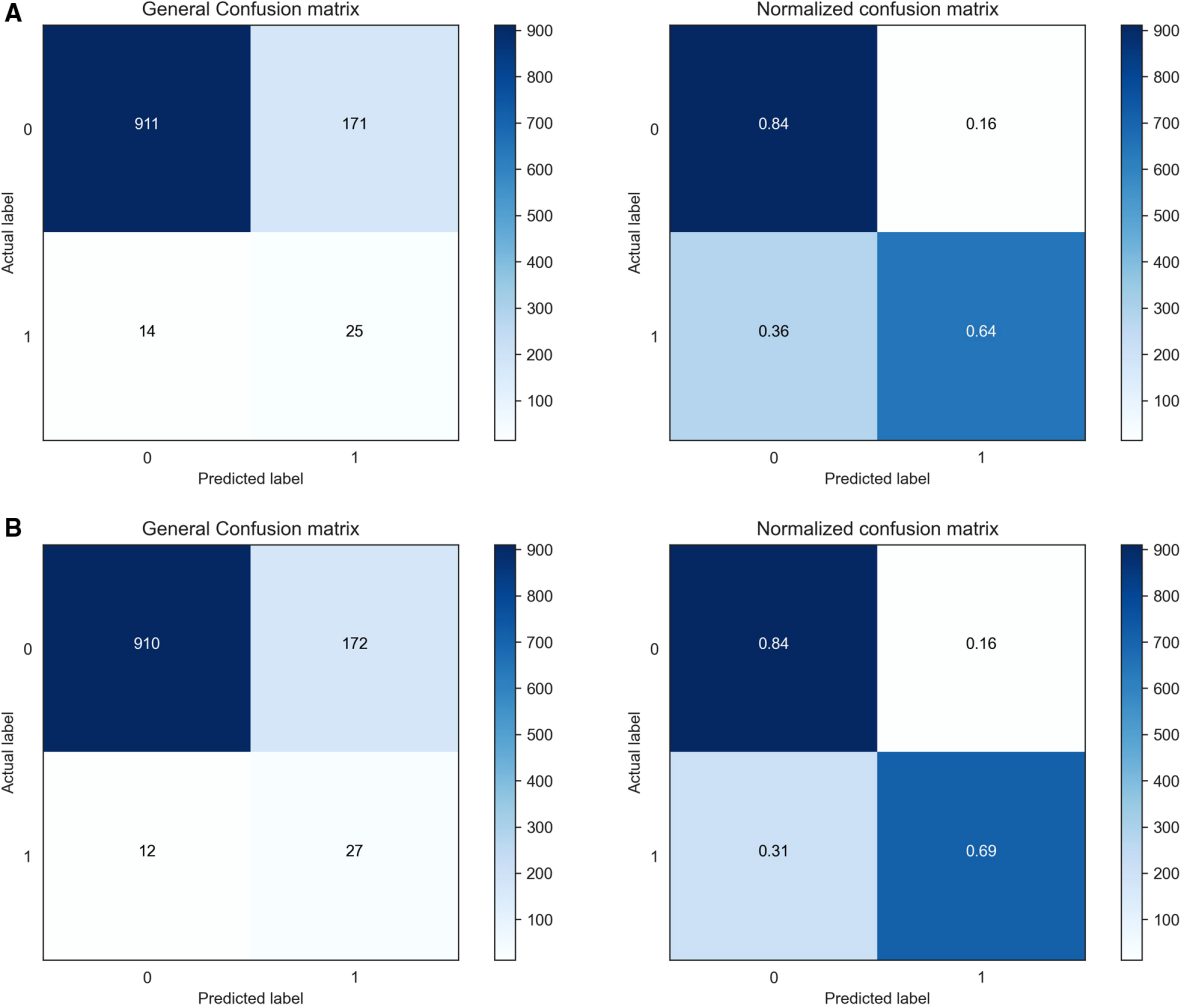
Confusion matrices and normalized confusion matrices of the proposed CSDNN model with the feature selection. (**A**) without threshold moving; (**B**) with threshold moving.

The confusion matrices of the proposed CSDNN model using the optimal features with and without moving the threshold were shown in Figure 6, where Figure 6A showed the confusion matrix and normalized confusion matrix of the proposed model without shifting the threshold and Figure 6B with moving the threshold. The figure shows the predicted class labels on the x-axis and the actual class labels on the y-axis, as well as 0 and 1 representing the survived and deceased patients, respectively. In the confusion matrix, the higher value for (0, 0) and (1, 1) indicates the more accurate prediction model for mortality in out-of-hospital AMI patients with hypertension. As a result, Figure 6A showed that the proposed CSDNN model without adjusting the threshold could predict 84% of the survived (*N* = 911) and 64% deceased (*N* = 25) patients accurately from the total population. The proposed model with moving the threshold was able to accurately predict 84% of the survived (*N* = 910) and 69% deceased (*N* = 27) patients as shown in Figure 6B, which was much higher than the result without applying the threshold moving technique.

### 4.3 Discussions

The mortality of CVD is continuously increasing every year globally and is strongly influenced by hypertension (3, 7). Early detection and management of people at risk before their symptoms appear is important. DL approaches have shown high performance in different domains, including healthcare (20–26), etc. Therefore, this paper was motivated to propose an accurate mortality prediction model for out-of-hospital Korean AMI patients with hypertension. In the experiment, the real-world AMI patients' dataset named KAMIR-NIH was used with 2-year follow-ups.

Since the experimental data was imbalanced, the cost-sensitive learning technique was performed in the proposed method. The effectiveness of the proposed model was proved by comparing it with other state-of-the-art ML and classic data sampling-based ensemble models. The results showed that the proposed CSDNN model could achieve better performance than all compared models. The cost-sensitive learning method could also improve the performance in most compared models such as LR, SVM, XGBoost, AdaBoost, etc. It also indicated that the cost-sensitive learning technique was a good solution to solve the class imbalance problem in the experimental data, which is supported by (30). In addition, optimizing the class weight value could also increase the final decision performance of the proposed model, and this result is consistent with a previous study (82). Figures 4E, G demonstrated that the proposed model achieved the best AUC of 0.7667 with the optimal class_weight value, which increased the AUC by 2.58% than the best performance of the state-of-the-art ML model AdaBoost and the AUC by 2.55% than the highest performance of the classic data sampling-based ensemble model EasyEnsemble for the mortality prediction of out of hospital AMI patients with hypertension.

Moreover, the performance of the proposed method was also improved by using the probability threshold moving technique. The results showed that the performance increased by about 3.5% of the proposed CSDNN model from the AUC of 0.7317–0.7667. It demonstrated the effectiveness of the threshold moving technique for the class imbalance problem, which is consistent with (35). However, the DT model with the cost-sensitive learning, and with and without shifting the threshold showed lower performance using the original features as well as the optimal extracted features. The reason for this is that the DT algorithm was proposed to predict the class correctly instead of the probability estimation (55). Additionally, the efficiency of developing the proposed model and other compared models was increased by using the RFE wrapper-based feature selection method, and the performances of the proposed model and many compared models such as LR, AdaBoost, etc. were also improved. The automatically selected 27 optimal features have been used as important risk factors related to the prediction of CVD patients in different studies (18, 41). The outcome can be a point of reference for various considerations by clinical experts for CVD prediction.

Several classic data sampling-based ensemble methods such as balanced bagging, balanced RF, EasyEnsemble, and RUSBoost were developed for the classification of imbalanced data. The proposed model which integrated the DNN, cost-sensitive learning, and threshold moving technique achieved better prediction performance than those methods. The current research established that it was also best practice to think about integrating various techniques for better prediction improvements. Finally, the proposed CSDNN model can be used as an aided diagnosis system for decision support in the mortality prediction of out-of-hospital AMI patients with hypertension.

However, there are several potential limitations in this paper. First, the result of this paper may not be suitable for patients from other populations due to the use of the Korean AMI dataset. Second, the proposed prediction model may not provide good performance for in-hospital patients or patients with short-term follow-ups since the experimental dataset used in this research was with 2-year follow-ups. Third, the experimental dataset was insufficient for the DL models since the DL models are data-hungry, and we could not collect more data. Moreover, the DL model was opaque even though it showed better results than other models.

## 5 Conclusion

In this paper, a CSDNN-based mortality prediction model was proposed for out-of-hospital Korean AMI patients with hypertension based on the real-world KAMIR-NIH dataset with 2-year follow-ups on imbalanced data. It was worthwhile to apply the cost-sensitive learning technique to overcome the imbalanced data problem and use the threshold moving technique to enhance the performance while using the feature selection method to increase efficiency. The results of our experiment showed that the proposed model outperformed other ML-based models and classic data sampling-based ensemble models with an AUC of 2.58% and 2.55% improvement over the best state-of-the-art ML model and the classic data sampling-based ensemble model, respectively. It is also expected that the results of this research will be useful for the decision-making of mortality prediction in AMI patients with hypertension. In the future, it is expected to collect more datasets from different countries to design an accurate and explainable mortality prediction model for multiple races.

## Data Availability

Publicly available datasets were analyzed in this study. This data can be found here: The experimental datasets are confidential and available with the permission of the Korea Acute Myocardial Infarction Registry (KAMIR, http://kamir6.kamir.or.kr/) on reasonable request.
